# Xyloglucan is released by plants and promotes soil particle aggregation

**DOI:** 10.1111/nph.14897

**Published:** 2017-11-15

**Authors:** Andrew F. Galloway, Martin J. Pedersen, Beverley Merry, Susan E. Marcus, Joshua Blacker, Liane G. Benning, Katie J. Field, J. Paul Knox

**Affiliations:** ^1^ Centre for Plant Sciences Faculty of Biological Sciences University of Leeds Leeds LS2 9JT UK; ^2^ School of Earth & Environment University of Leeds Leeds LS2 9JT UK; ^3^ German Research Centre for Geosciences GFZ Potsdam 14473 Germany; ^4^ Department of Earth Sciences Free University of Berlin Berlin 14195 Germany

**Keywords:** cereals, glacial forefields, liverworts, plant roots, polysaccharide, rhizoids, soil aggregates, xyloglucan

## Abstract

- Soil is a crucial component of the biosphere and is a major sink for organic carbon. Plant roots are known to release a wide range of carbon‐based compounds into soils, including polysaccharides, but the functions of these are not known in detail.
- Using a monoclonal antibody to plant cell wall xyloglucan, we show that this polysaccharide is secreted by a wide range of angiosperm roots, and relatively abundantly by grasses. It is also released from the rhizoids of liverworts, the earliest diverging lineage of land plants. Using analysis of water‐stable aggregate size, dry dispersion particle analysis and scanning electron microscopy, we show that xyloglucan is effective in increasing soil particle aggregation, a key factor in the formation and function of healthy soils.
- To study the possible roles of xyloglucan in the formation of soils, we analysed the xyloglucan contents of mineral soils of known age exposed upon the retreat of glaciers. These glacial forefield soils had significantly higher xyloglucan contents than detected in a UK grassland soil.
- We propose that xyloglucan released from plant rhizoids/roots is an effective soil particle aggregator and may, in this role, have been important in the initial colonization of land.

## Introduction

Soil is a critical component of the terrestrial biosphere. It plays a pivotal role in geochemical cycling of carbon and nutrients, which subsequently drives terrestrial ecosystem composition, function and the long‐term regulation of global climate (Paustian *et al*., 2016; Leake & Read, 2017). Soils are known to possess a carbon storage capacity exceeding that of the above‐ground biomass, making them a major contributor to terrestrial carbon cycling (Scharlemann *et al*., 2014; Lehmann & Kleber, 2015; Paustian *et al*., 2016). As such, soil is responsible for many of the ecosystem services that are essential to human life, including agriculture, water and atmospheric gas composition. Declining soil health and function as a result of changes in climate and human land use is a major global problem, presenting significant challenges to future food and water security (Lal, 1997; Kibblewhite *et al*., 2008). Soil degradation is marked by reduced soil particle aggregation with associated increased compaction and reduced water flow. Organic matter of plant, animal or microbial origin underpins many soil properties, such as water‐holding capacity, porosity, microbial community composition and fertility, although it can be challenging to dissect beyond total or organic carbon content (Lehmann & Kleber, 2015). Furthermore, the roles of soil organic matter (SOM) in maintaining particle aggregation that underpins soil properties in relation to water holding and microbial communities are not well defined.

Polysaccharides of microbial and plant origin are implicated in the maintenance of soil aggregation and properties (Tisdall & Oades, 1982; Cheshire & Hayes, 1990; Oades, 1993), although their molecular identities and precise functions remain unknown. Plant roots are known to contribute to the total organic carbon content of soils, through their own polysaccharide‐rich biomass and also through secreted carbon‐based molecules in mucilages and signalling compounds (Walker *et al*., 2003; Dennis *et al*., 2010; Baetz & Martinoia, 2014). Analyses of polysaccharide components of plant root mucilage generally reflect cell wall polysaccharides, and major components reported are pectic polysaccharides and arabinogalactan‐proteins (Bacic *et al*., 1986; Moody *et al*., 1988; Knee *et al*., 2001; Driouich *et al*., 2013). However, relatively few studies have been done and precise identities of polysaccharides released by plants into soils and any role(s) they play in rhizospheres and wider soil environments remain largely unknown, although they are proposed to have roles in improving root penetration, soil properties and influencing soil microbial communities (York *et al*., 2016; Zickenrott *et al*., 2016).

Monoclonal antibodies directed to cell wall polysaccharides are highly sensitive and versatile molecular tools for the detection and assessment of polysaccharides in various contexts. Using the LM25 xyloglucan monoclonal antibody (Pedersen *et al*., 2012), we have identified xyloglucan as a polysaccharide released by both liverwort rhizoids and plant roots and found it to be released by all plants surveyed. This led us to explore the potential roles of xyloglucan in soil environments and to identify it as a potent soil particle aggregator.

## Materials and Methods

### Plant materials

Wheat (*Triticum aestivum* L. cv Cadenza), maize (*Zea mays* L. cv Earlibird), barley (*Hordeum vulgare* L. cv Golden Promise), pea (*Pisum sativum* L. cv Avola), tomato (*Solanum lycopersicum* L. cv Ailsa Craig) and rapeseed (*Brassica napus* L. cv Extrovert) plants were grown hydroponically for 14 d (after 7 d for seedling establishment in Perlite) with eight seedlings in 9 l volumes in half‐strength Hoagland's solution. *Arabidopsis thaliana* (L.) Heyn. ecotype Col‐0 seedlings were grown for 14 d and the moss *Physcomitrella patens* (Hedw.) Bruch & Schimp., and liverworts *Marchantia polymorpha* L. and *Blasia pusilla* L. were all grown in continuous shaking liquid culture with 4–6 wk between subcultures with BG11 medium (Rippka *et al*., 1979), as described for *B. pusilla* (Jackson *et al*., 2012). In all cases, plant FWs were taken at the point of medium collection and xyloglucan contents were determined by immunoassay. In some cases, liverworts (*M. polymorpha* and *Lunularia cruciata* L.) were collected locally and gemmae were taken from thallus cups and immediately placed on solid 1% (w/v) agar with water and maintained in a moist atmosphere under low light. After the time points, gemmae and all rhizoids were removed and 1 ml volumes of agar centred on former positions were removed with a cork borer and excised gel pieces were diced and incubated with 1 ml of water overnight. The water extracts were used for analyses. In additional analyses, nitrocellulose prints of agar surfaces after removal of gemmae were prepared by laying nitrocellulose sheets on agar surfaces for 30 min before sheet removal for processing. *A. thaliana* seedlings were also grown on solid media (Cornuault *et al*., 2014) and solid media surfaces were printed onto nitrocellulose for similar immunoanalyses.

### Xyloglucan assays with monoclonal antibody LM25

Initial screening of plant hydroponates and growth media involved enzyme‐linked immunosorbent assays (ELISAs) of materials coated directly onto microtitre ELISA plates (Supporting Information Tables S1, S2). For subsequent ELISA quantification of xyloglucan in hydroponates*,* shaking culture plant media, solid growth media water extracts and soil alkali extracts, solubilized materials were titrated fivefold onto microtitre plates and incubated in a high salt buffer to ensure efficient microtitre plate well coating overnight before processing with rat monoclonal antibody LM25 to detect xyloglucan (Pedersen *et al*., 2012). Sample titrations were extended as appropriate to ensure absorbance readings in the range 0–1.0 OD (optical density) which was used to generate xyloglucan equivalents using tamarind xyloglucan (Megazyme International, Bray, Ireland) as a standard. Nitrocellulose prints of solid growth media surfaces were developed with LM25 at 10‐fold dilution followed by anti‐rat immunoglobulin G horseradish peroxidase as previously described (Willats *et al*., 1998). *In situ* immunofluorescence analysis of *M. polymorpha* gemmae was performed with gemmae in agar plugs incubated in volumes of antibody solutions using standard indirect labelling methods (Jackson *et al*., 2012).

### Soil particle aggregation analyses

Wet sieving was performed on 100 g samples of sterilized sandy loam with an addition of the representative polysaccharides, tamarind seed xyloglucan, polygalacturonic acid (Megazyme International) and gum Arabic (Sigma‐Aldrich). Polysaccharides were added dry to moist sandy loam samples and thoroughly mixed, and then 100 ml of water was added to each sample and mixing continued for 2 h before wet sieving analysis. Wet sieving used an Octagon mechanical shaker (Endecotts Ltd, London, UK) set at an amplitude of 1.8 mm with a constant flow of tap water, which produced five size fractions of soil aggregates: > 1000, 500–1000, 250–500, 90–250 and < 90 μm. Sieves were placed at 95°C for 30 min and then transferred to preweighed blotting card envelopes and placed at 40°C overnight before weighing.

Dry dispersion particle analysis was carried out using a Morphologi G3 (Malvern Instruments Ltd, Malvern, UK) particle characterization microscope. In this case, 5 g of sandy loam was mixed with test polysaccharides already dissolved in 10 ml of distilled water and mixed for 2 h. After mixing, samples were centrifuged for 10 min at 3856 ***g***. Samples of pelleted soil were dried for 48 h at 40°C. Soil samples (18 mm^3^) were added into the dispersal unit which dispensed samples onto a glass slide with an injection pressure of 1 bar. Before and after each analysis, the dispersal unit and blast chamber were cleaned with antistatic spray. The Morphologi G3 imaged 50 000 particles per replicate using a ×5 objective.

Scanning electron microscopy (SEM) was used to study dried samples as prepared for particle analysis and was carried out using a Quanta 200 F scanning electron microscope (Thermo Fisher Scientific, Loughborough, UK). A thin layer of soil was spread onto glass and stubs with carbon‐rich tape were dipped into the soil and then immediately coated with a 5‐nm‐thick layer of platinum under a vacuum before SEM imaging.

### Soil sampling, total organic carbon (TOC) and xyloglucan analyses

A sandy loam for soil aggregate analyses was obtained locally in May 2015. All visible plant material was removed from the sampled soils and these were sieved using a 2 mm analytical sieve and sterilized by autoclaving spread on a metal tray and then stored in the dark at 4°C until use. Farm grassland soil samples were obtained from a permanent pasture (GPS coordinates 53.872/−1.323) in February 2016. Developing soils were collected in June 2014 from two subArctic glacier forefields on the east and western sides of the Kebnekaise massif in northern Sweden (Storglaciären, 67°54′N, 18°34′E; and Rabots, 67°55′N, 18°29′E) and from a high‐Arctic glacier forefield in Svalbard, Norway (Midre Lovénbreen, 78°53′N, 12°3′E) in August 2013. The proglacial soil ages were estimated using aerial photography to be *c*. 80 and 104 yr for Storglaciären and Rabots, respectively, while that of the Midtre Lovenbreen soils was *c*. 2000 yr based on radio carbon dating (Hodkinson *et al*., 2003). In each case three independent samples of both farm and glacial forefield soils from the same vicinity and from the upper 15 cm of soil were analysed. All visible plant materials were discarded at the time of collection, and the soils were dried and sieved (< 2 mm). One gram each of these soils was extracted with 2.5 ml of 4 M KOH for 1 h and then neutralized with 80% acetic acid and the extracts were stored at 4°C until use. TOC was analysed in all grassland and glacial forefield soils after crushing the sieved fractions to < 63 μm and treating the powders overnight with HCl to remove inorganic carbonates; these samples were combusted at 1350°C using a LECO SC 144DR elemental analyser (Leco Corp., St Joseph, MI, USA) calibrated with certified reference materials; analytical precision was < 5% and the limit of detection of 0.0062 g kg^−1^.

## Results

### Xyloglucan is released by a wide range of land plants

**Figure 1:**
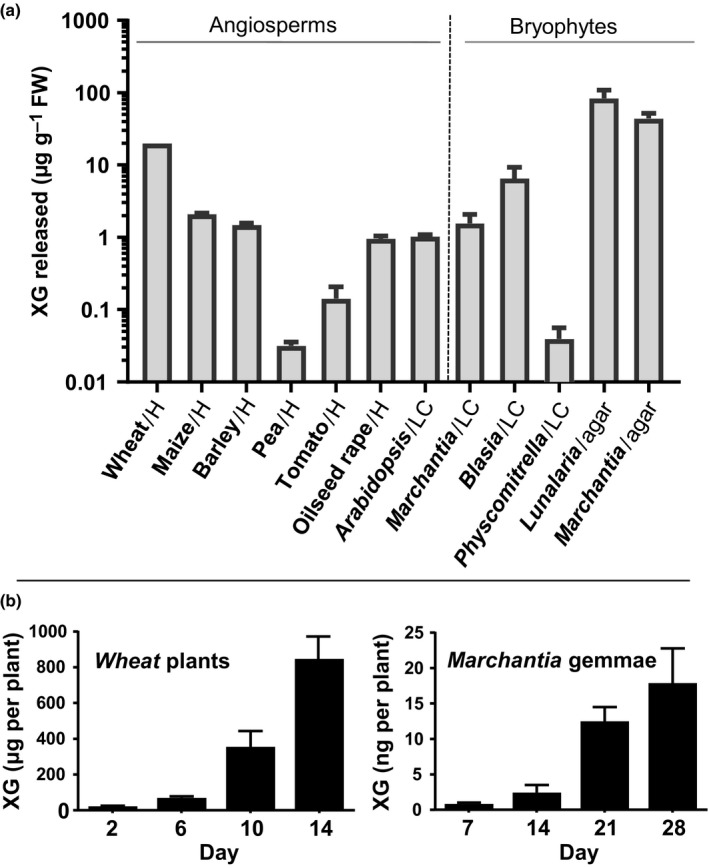
Xyloglucan (XG) secretion from plants as determined by enzyme‐linked immunosorbent assay (ELISA) with XG MAb LM25 (commercial XG equivalents). (a) Survey of XG release from a range of angiosperms and bryophytes. Most angiosperms were grown in a hydroponic system (H) for 14 d and FW and XG in hydroponate were assessed. *Arabidopsis* and bryophytes were grown in shaking liquid culture (LC) for 28 d and FWs and XG in media were assessed. In the case of *Marchantia polymorpha* and *Lunularia cruciata*, gemmae were grown on solid media (agar, water) for 28 d and the gemmae were removed and agar extracted for ELISA assessments. Results are presented as XG (g g^−1^ FW). *n *=* *3; error bars, + SD. (b) Wheat seedlings grown hydroponically (eight seedlings, aerated 9 l medium) and growth medium samples taken at indicated time points for ELISA assessment. *M. polymorpha* gemmae were placed individually on agar solid media and at time points gemmae were removed, and agar plugs were taken and extracted with 1 ml water for ELISA assessments. Results are presented as XG released per plant at shown time points. Error bars, + SD.

The polysaccharide components released from plant roots have not been characterized extensively. To initiate a programme to enhance the understanding of the structures and functions of plant polysaccharides that enter rhizospheres and soil environments, panels of monoclonal antibodies directed to plant cell wall polysaccharides (including pectic polysaccharides and arabinogalactan‐proteins) were used to assay the contents of growth media subsequent to plant growth (Tables S1, S2). These analyses included the media of six eudicotyledon species grown hydroponically and also the media of *A. thaliana* and three bryophytes grown in shaking liquid culture. An unexpected feature common to all these growth media was the detection of xyloglucan, as determined by LM25 immunoassay. The LM25 xyloglucan epitope was detected particularly strongly in media of three grass species, whereas pectic polysaccharides were only weakly detected (Fig. 1; Table S1), which was of interest, as grass species such as wheat, maize and barley have a relatively low concentration of xyloglucan within cell walls (Vogel, 2008). Xyloglucan was also found to be released into the media of bryophytes that do not have roots but soil‐penetrating rhizoids and in this case xyloglucan was the major polysaccharide detected in bryophyte growth media (Table S2).

As xyloglucan release by plants was detected across the land plant phylogeny and abundantly in secretions of both cereals and liverworts, it was decided to focus our studies on this polysaccharide. Xyloglucan was found to be particularly abundant in the media of liverwort species when expressed on a per‐unit‐FW basis (Fig. 1). The time course of xyloglucan released by wheat seedlings growing hydroponically and *M. polymorpha* gemmae grown on agar is shown in Fig. 1 and indicates that for both of these highly diverged species, xyloglucan steadily accumulates in the media during growth. It was found that wheat grown hydroponically for 14 d released in the region of 800 μg xyloglucan per plant (Fig. 1), which is equivalent to *c*. 20 μg g^−1^ FW, and that more than 15 ng of xyloglucan was released from each *M. polymorpha* gemma cultured for 28 d on solid agar media (quantified by extractions of media after gemmae removal; Fig. 1). This is equivalent to > 50 μg g^−1^ FW, making the amount of xyloglucan secreted from *M. polymorpha* rhizoids comparable to or greater than that released by wheat roots grown in a hydroponic system.

**Figure 2:**
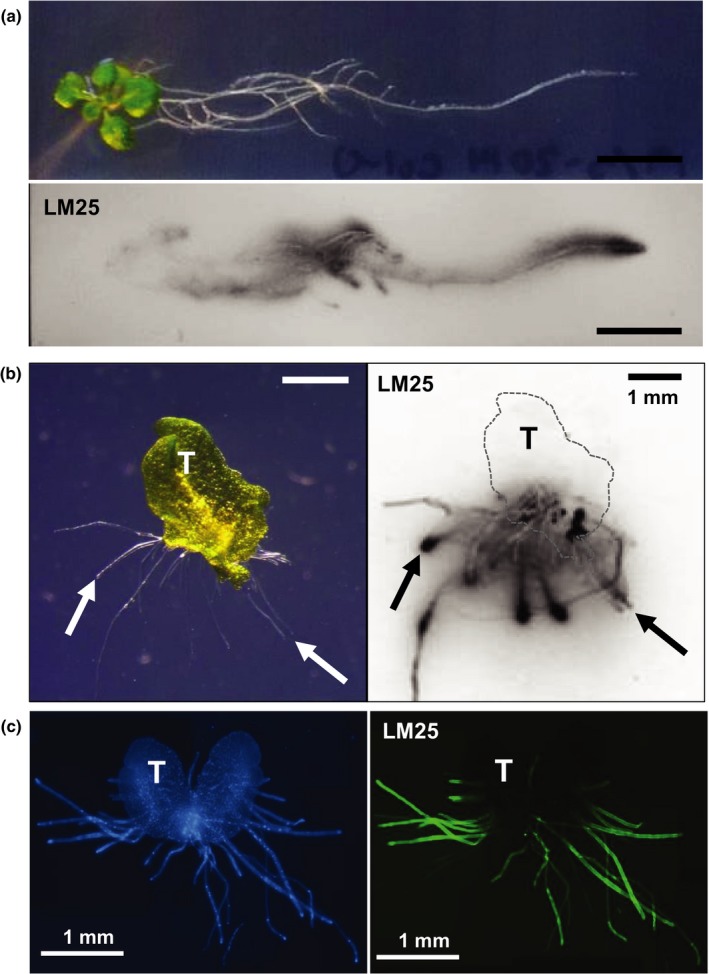
Detection of xyloglucan (XG) secretion from plant surfaces with XG MAb LM25. (a) Bright field image of *Arabidopsis* seedling grown on plant agar solid media for 14 d paired with nitrocellulose print of solid media surface (after removal of the seedling) which was then probed with LM25. Bars, 10 mm. (b) Secretion by day‐30 *Marchantia polymorpha* gemma. Bright field image on agar and immunoprint of agar surface after gemma removal. Arrows indicate corresponding rhizoid tips. T, thallus outlined in print by dashed line. Bars, 1 mm. (c) Whole mount immunolabelling of day‐14 *M. polymorpha* thallus/rhizoid *in situ* on agar block with LM25. Blue represents Calcofluor White labelling of cell walls and green represents LM25‐FITC. T, thallus. Bars, 1 mm.

The versatility of monoclonal antibodies allows the spatial assessment of released xyloglucan on the nitrocellulose prints of solid media growth surfaces after removal of plants, as shown for an *A. thaliana* seedling and a *M. polymorpha* gemma (Fig. 2). In the case of *A. thaliana* root, xyloglucan is abundant at the tips and appears to be diffuse, indicating high solubility in the solidified medium and not a tight association with root tip mucilage, and this is also true for *M. polymorpha* rhizoids where immunoprints unequivocally indicate the release of xyloglucan from the rhizoids and not the thallus (Fig. 2). Furthermore, the rhizoids are strongly and specifically labelled with the LM25 xyloglucan probe, as determined by immunofluorescence in a whole mount approach (Fig. 2).

### Xyloglucan is an effective promoter of soil aggregation

**Figure 3:**
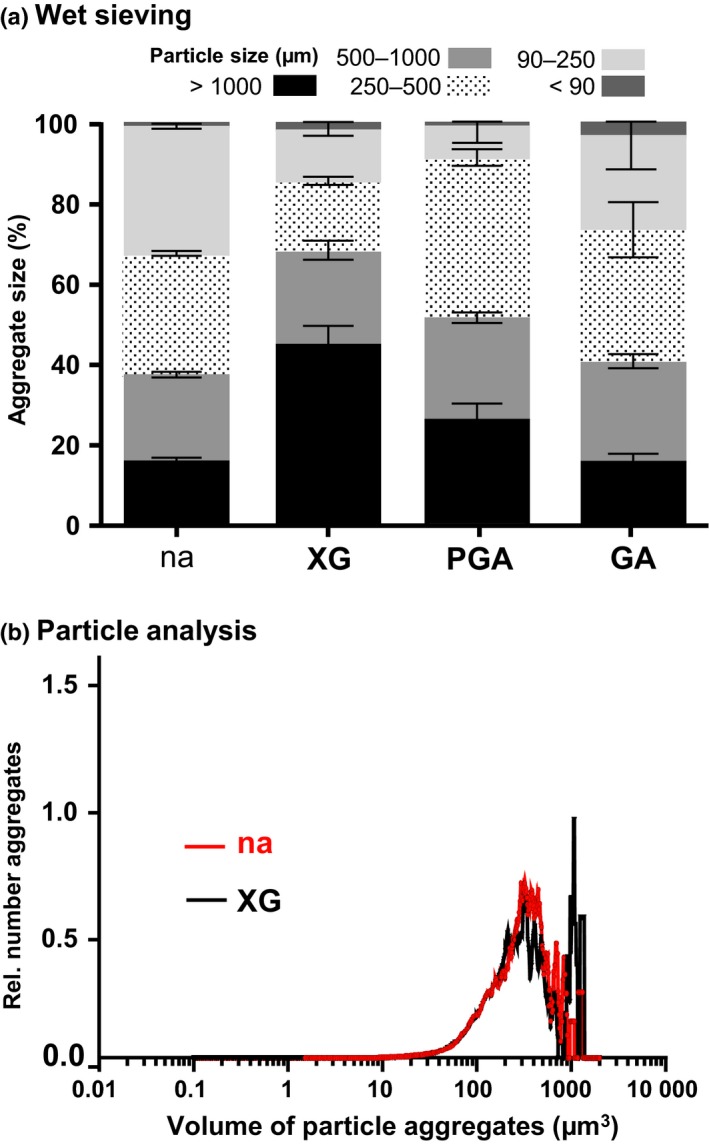
Xyloglucan (XG) impact on soil aggregation. (a) Wet sieving analyses of a sterilized sandy loam with nothing added (na), with addition of tamarind seed XG, polygalacturonic acid (PGA) and gum Arabic (GA; all at 1% (w/w), 10 g kg^−1^). Error bars, ± SD. (b) Automated particle analysis after dry dispersion using a Morphologi G3 microscope. Data showing distribution of particle volumes with no addition (na) and 0.1% (w/w), 1 g kg^−1^ XG.

**Figure 4:**
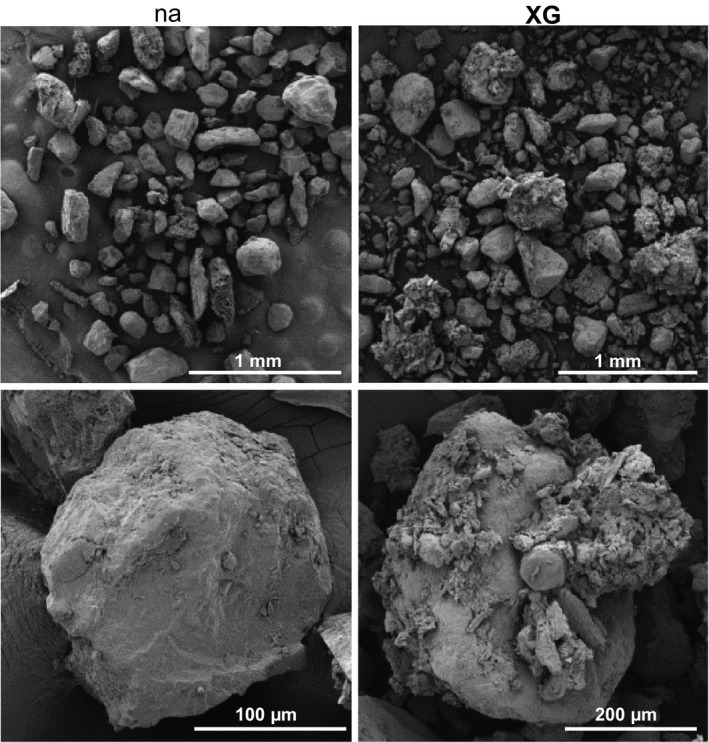
Scanning electron micrographs showing xyloglucan (XG) impact on aggregation of sandy loam particles without (na) and with XG at 0.1% (w/w), 1 mg g^−1^ soil.

To elucidate possible roles and the influence of soluble xyloglucan in the rhizosphere and on soil properties, commercially available xyloglucan was added to a sterilized sandy loam (1% (w/w), 10 g kg^−1^). It was found to be effective in promoting the aggregation of soil particles (Fig. 3), being even more effective in soil aggregation than the pectic polysaccharides and arabinogalactan‐proteins detected in root mucilage (Fig. 3). Wet sieving indicated that when xyloglucan was added, the weight proportion of water‐stable soil aggregates > 1000 μm doubled from < 20% to > 40% of aggregates and the proportion < 250 μm halved from > 30% to *c*. 15% of all aggregates on a per‐weight basis (Fig. 3). This capacity of xyloglucan to promote soil particle aggregation was confirmed using automated particle characterization after dry sample dispersion with a Morphologi G3 automated imaging system. This showed a significant increase in aggregate volumes > 2000 μm^3^ as a result of addition of xyloglucan at 0.1% (w/w) (i.e. a 10 times lower concentration than in the wet‐sieving analysis) to the sandy loam (Fig. 3). The influence of xyloglucan on soil structures was visualised using SEM, which revealed a distinctive occurrence of larger particles being coated with smaller soil particles and also a clear indication of increased attachment of smaller soil particles to the substrate (Fig. 4).

### Xyloglucan can be extracted from soils by alkali treatments and is relatively abundant in glacial forefield soils

**Figure 5:**
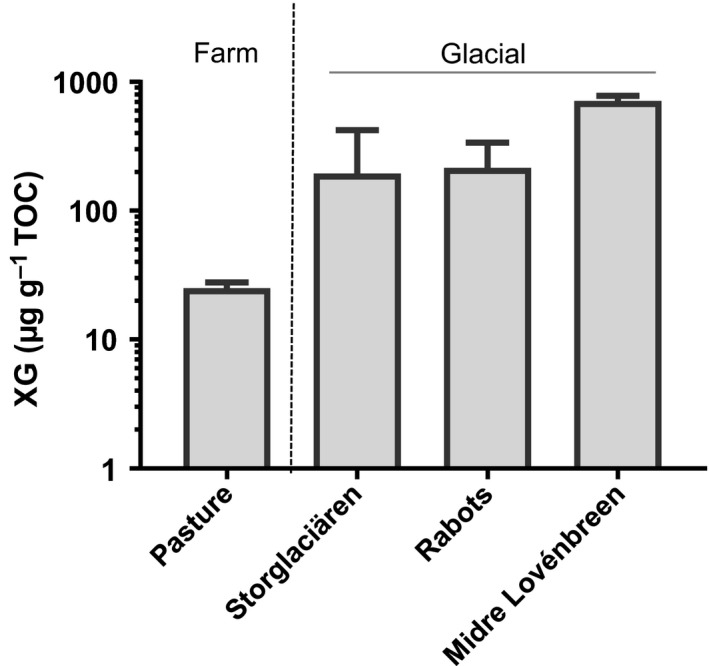
Xyloglucan (XG) contents expressed as μg g^−1^ soil total organic carbon (TOC) in a sample of UK farm grassland soil (pasture) and three glacial forefield soil samples as determined by KOH extraction. Error bars, + SD,* n *=* *3. Soils from Storglaciären and Rabots glaciers are *c*. 120 yr old (classed as regosols) with, in all cases, TOC < 6 g kg^−1^ soil; that of Midre Lovénbreen glacier is *c*. 2000 yr old (a cryosol) with TOC of 29.4 ± 0.8 g kg^−1^; and the grassland soil TOC is 36.0 ± 2.9 g kg^−1^.

Liverworts are important in the colonization of soils, both in terms of modern‐day environments and in the processes on the early Earth during land colonization by plants (Field *et al*., 2015; Mitchell *et al*., 2016). To extend our understanding of how plant‐secreted xyloglucan, particularly that from liverworts, may influence the formation of soils, we explored the xyloglucan content of young proglacial soils from Arctic Sweden and Norway. We found that alkali extractions of proglacial soils release significant amounts of xyloglucan compared with a typical UK grassland soil (Fig. 5). The TOC contents of the Storglaciären and Rabots forefield soils (both *c*. 100 yr old and classed as regosols) were 3.46 ± 1.62 and 0.89 ± 0.36 g kg^−1^ soil, respectively. The TOC of the *c*. 2000‐yr‐old soil from the Midre Lovénbreen forefield (a cryosol) was an order of magnitude higher at 29.4 ± 0.8 g kg^−1^, and similar to the TOC of the grassland soil (36.0 ± 2.9 g kg^−1^). When expressed on a xyloglucan/TOC basis, there was a striking abundance of xyloglucan in all three glacial forefield mineral soils and particularly for the *c*. 2000‐yr‐old soil which had > 700 μg g^−1^ TOC (Fig. 5). In this proglacial soil, xyloglucan was detected at concentrations of > 20 mg kg^−1^ soil. The equivalent analyses of the UK grassland soil indicated a xyloglucan content of *c*. 0.8 g kg^−1^ soil.

## Discussion

To date the focus on xyloglucan in plant biology has been on its role in plant cell walls where it is a major matrix polysaccharide that is particularly abundant in the primary cell walls of eudicots (*c*. 20% wall polysaccharides) but less so in grass cell walls (*c*. 5%) (Vogel, 2008; Park & Cosgrove, 2015). Xyloglucan is a branched polysaccharide with a β1,4‐glucan backbone with side chain structures including xylosyl, galactosyl and fucosyl residues (Park & Cosgrove, 2015). In the context of cell wall matrices, xyloglucan's roles in binding to and tethering cellulose microfibrils and in the control of cell expansion remain an active area of research (Park & Cosgrove, 2015).

Our extended analyses indicate that xyloglucan secretion is a widespread phenomenon across land plants with particular abundance in cereal hydroponates and also, at the other end of land plant phylogeny, in liverwort growth media. Liverworts do not possess roots, but instead produce extensive rhizoids – tip‐growing cellular extensions in some ways comparable to root hairs (Jones & Dolan, 2012; Duckett *et al*., 2014) with conserved mechanisms of cell wall formation (Honkanen *et al*., 2016). The cellular basis of the release of xyloglucan by plant roots – whether root tips or root hairs or both – remains to be determined. The significance of the apparently relatively low concentrations of xyloglucan released by pea is also unclear at this stage. It is possible that the release of factors from root hairs may be different in legumes in the context of root hairs as points for *Rhizobia* entry into roots. It is of interest in this context that in *A. thaliana*, a root hair‐specific acidic xyloglucan (with galacturonic acid as a side chain residue) has been identified and also that a structurally similar acidic xyloglucan has been identified in liverwort cell walls (Peña *et al*., 2008, 2012). Additionally a related xyloglucan‐specific galacturonosyltransferase has roles in both root hair and rhizoid development (Honkanen *et al*., 2016). It is also of interest here that, in this initial survey, cultured *P. patens* secretes relatively low concentrations of xyloglucan in comparison with the liverwort species. In liquid cultures, *P. patens* mainly produces chloronemata which are very different from liverwort rhizoids (Pressel *et al*., 2008). Indeed, the multicellular filament systems (rhizoids, caulo‐ and chloronemata) found in mosses may well have had a separate evolutionary origin as these attributes are absent in basal moss clades (Newton *et al*., 2000). However, there has been no previous report of the abundant release of xyloglucan by land plants other than the identification of xyloglucan structural features in maize and wheat root mucilage (Bacic *et al*., 1986; Moody *et al*., 1988). Similarly, there has been little previous work on factors released from rhizoids (Odu, 1989) and it was once thought that their major role was anchorage, but now it is clear they are likely to be involved in nutrient acquisition and associations with fungi (Pocock & Duckett, 1985; Duckett *et al*., 2014).

The work presented here has targeted soluble molecules that are released from roots/rhizoids and which may not be features of the structurally coherent mucilage that stays adhered to root tips. Future work will need to define the structures of these released glycans and also the cellular and molecular mechanisms of release from roots and rhizoids.

### Impacts of released xyloglucan in rhizospheres

There have been only a few studies of the effect of exogenous polysaccharides on soil properties such as compressive strength and hydration characteristics and these have included xanthan and dextran of bacterial origin and polygalacturonic acid (Czarnes *et al*., 2000; Hart *et al*., 2001; Chang *et al*., 2015). Here, using proxies of three polysaccharides known to be detected in plant mucilage and in plant growth media, we show that xyloglucan is the most effective in increasing the proportion of large aggregates in a sandy loam. Future work will require the assessment of isolates of actual released polymers, and also a wider consideration to include microbial polysaccharides, to develop a full understanding of polysaccharide impacts on soil aggregate status. The soil supplementation studies reported here have used much higher concentrations of xyloglucan than were detected in our bulk analyses of soil. However, we propose that the local concentrations of xyloglucan and other plant polysaccharides in the immediate rhizosphere surrounding the plant root/rhizoid surfaces are likely to be of a much higher concentration than those indicated by our bulk soil analyses; indeed, it is possible that they accumulate in soils in these areas influencing rhizosphere properties.

Factors such as xyloglucan may be key in driving improved local soil structure, including soil particle adhesion and enhanced soil water‐holding capacity (Dennis *et al*.,^2010^; Carminati & Vetterlein, 2013; York *et al*., 2016). An additional factor here, in the disparity in the amounts detected in bulk soils and the concentrations of added xyloglucan required to demonstrate a change in aggregate status by wet sieving analysis, dry dispersion and SEM techniques, is that we do not yet know the biochemical natures of the xyloglucans released by land plants and these are likely to be different from tamarind seed xyloglucan used in our studies. This commercial form of xyloglucan has no acidic residues that are known to be present in xyloglucan of *A. thaliana* roots and liverworts (Peña *et al*., 2008, 2012), as discussed earlier. It is possible that specific biochemical forms of released factors have different properties and secreted xyloglucan may be more effective in soil particle adhesion than the form of xyloglucan we have used to date. Moreover, the extent of release of polysaccharides in relation to plant biomass may be very different between plants grown in soil and those grown in hydroponic and culture systems. The root systems within a volume of soil can be extensive (Dittmer, 1937) and a large root–soil interface is likely to have an associated large capacity to secrete exudates, including xyloglucan. Further work should focus in this area and aim to characterize not only the identity and structures of plant root/rhizoid‐secreted polysaccharides, but also the effects of such secretions on surrounding microbial communities, particularly in terms of community structure and functioning. Furthermore, investigations are also now needed to define the responses of plant root/rhizoid secretions to variation in growth environments and nutrient availabilities.

### Plant terrestrialization and first soils

Given the critical placement of liverworts at the base of the extant land plant phylogeny (Ligrone *et al*., 2012), our finding that they secrete xyloglucan from their rhizoids is evolutionarily significant. Xyloglucan release has been detected across the complex thalloid liverwort phylogeny, including both early divergent genera (*Blasia* and *Lunularia*) and the later divergent *Marchantia* genus (Crandall‐Stotler *et al*., 2009). The abundant release of xyloglucan by *B. pusilla* may relate to it being a known colonizer of newly exposed mineral soils (Mitchell *et al*., 2016), and extended studies of xyloglucan secretion by liverworts in the context of ecology are likely to be fruitful. Analysis of genes encoding proteins involved in xyloglucan biosynthesis and metabolism are indicative that this polysaccharide was an important structural feature of ancestral charophytes that mediated their colonization of land (Del Bem & Vincentz, 2010; Harholt *et al*., 2016). We propose that xyloglucan (or equivalent polysaccharide) secretions from the rhizoids or rhizoid‐like structures of the earliest land plants colonizing Earth's continental land masses > 475 million yr ago may have played a role in the formation of the first soils by modifying their immediate rhizospheric environments acting as a transient particle‐binding agent, aiding aggregation. Such alterations in primordial soils would facilitate SOM and carbon preservation, while contributing to increased aeration and water movement (Tisdall & Oades, 1982; Oades, 1993). This is likely to have had knock‐on effects in establishing early soil biotic communities and downstream impacts on mineral weathering and geochemistry (Lenton *et al*., 2012; Quirk *et al*., 2015). The turnover time of xyloglucan within soils is probably short as it is easily assimilatable by microbes. The relatively elevated xyloglucan contents of glacial forefield soils relative to a grass land soil may be a result of abundance of xyloglucan‐secreting plants, xyloglucan‐rich biomass and/or less developed microbial systems capable of xyloglucan degradation. The potential for polysaccharide recalcitrance to enzymatic degradation when adhered to mineral particle surfaces will be an interesting area for future study.

In summary, here we show that xyloglucan is widely secreted by plant roots, and also importantly by liverwort rhizoids, and has the capacity to play a role in soil structure and functioning through its action as a potentially transient particle‐binding agent. These findings strongly support the hypothesis that xyloglucan played a crucial role in plant terrestrialization and the formation of the first soils. This work extends our understanding of plant–soil biology and also presents new approaches to dissect polysaccharides as important factors influencing soil structures and properties.

## Author contributions

A.F.G., M.J.P., J.B., L.G.B., K.J.F. and J.P.K. planned and designed the research. A.F.G., M.J.P., J.B., B.M. and S.E.M. performed experiments, conducted field work, and analysed the data. A.F.G, J.B., L.G.B., K.J.F. and J.P.K. wrote the manuscript.

## Supporting information

Please note: Wiley Blackwell are not responsible for the content or functionality of any Supporting Information supplied by the authors. Any queries (other than missing material) should be directed to the *New Phytologist* Central Office.

**Table S1** Cell wall polysaccharide epitope detection in hydroponates from six crop species
**Table S2** Cell wall polysaccharide epitope detection in medium from liquid cultures of Arabidopsis and bryophytes
