# Supplementary material for: Xyloglucan is released by plants and promotes soil particle aggregation
Source: New Phytol. 2017 Nov 15;217(3):1128–36. doi: 10.1111/nph.14897 (PMC5813166; doi:10.1111/nph.14897)
Supplement: Supplementary file 1 — Table S1 Cell wall polysaccharide epitope detection in hydroponates from six crop species Table S2 Cell wall polysaccharide epitope detection in medium from liquid cultures of Arabidopsis and bryophytes [file NPH-217-1128-s001.pdf]

***New Phytologist* Supporting Information**

Article title: **Xyloglucan is released by plants and promotes soil particle aggregation**
Authors: Andrew F. Galloway, Martin J. Pedersen, Beverley Merry, Susan E. Marcus, Joshua Blacker, Liane G. Benning, Katie J. Field and J. Paul Knox
Article acceptance date: 18 October 2017

The following Supporting Information is available for this article:

Table. S1 Heat maps of cell wall polysaccharide epitope detection in hydroponates from six crop species. Plants were grown for two or three weeks and hydroponates immunoassayed directly by ELISA. Absorbance values shown are those for 1:25 dilution for the grasses and 1:5 dilution for pea, tomato and rapeseed analyses. Data are a mean of thee biological replicates. Values shown are absorbance units at 450 nm.

Antigen	**MAb**	Grasses			Eudicotyledons		
		Wheat	Barley	Maize	Pea	Tomato	Rapeseed
Pectic polysaccharides *Homogalacturonan*	LM19	0.10	0.11	0.12	0.18	0.3	0.2
	JIM7	0.06	0.1	0.08	0.15	0.91	0.12
	LM20	0.06	0.07	0.08	0.15	0.21	0.09
Rhamnogalacturonan-I	LM5	0.07	0.12	0.11	0.2	0.27	0.13
	LM6	0.06	1.58	0.08	0.17	0.72	0.09
Arabinogalactan-protein	LM2	0.78	0.71	0.33	0.33	2.83	0.13
	MAC207	0.05	0.09	0.09	0.16	1.08	0.73
Extensin	LM1	1.45	1.19	0.21	0.6	0.49	0.09
Xylan	LM11	1.13	0.69	0.39	0.21	0.29	0.6
Xyloglucan	LM25	2.32	1.14	1.25	0.29	0.25	0.57

Table. S2 Heat maps of cell wall polysaccharide epitope detection in medium from liquid cultures of Arabidopsis and bryophytes. Shaking liquid cultures of Arabidopsis (*At*) and three bryophytes: *Physcomitrella patens* (Pp), *Marchantia polymorpha* (Mp), and *Blasia pusilla* (Bp). *At* assay of 1:5 dilution of media and bryophytes 1:25 dilution of media. Values shown are absorbance units at 450 nm.

		Bryophytes		
**MAb**	*At*	*Pp*	*Mp*	*Bp*
LM19	0.52	0.07	0.08	0.12
JIM7	0.17	0.06	0.06	0.07
LM20	0.10	0.05	0.06	0.07
LM5	0.09	0.07	0.06	0.09
LM6	0.20	0.06	0.05	0.07
LM2	0.26	0.10	0.34	0.63
MAC207	0.15	0.15	0.15	0.33
LM1	0.89	0.05	0.06	0.37
LM11	0.15	0.06	0.07	0.08
LM25	0.65	0.42	0.41	1.84
